# Sphingosine 1-Phosphate Distribution in Human Plasma: Associations with Lipid Profiles

**DOI:** 10.1155/2012/180705

**Published:** 2012-11-08

**Authors:** Samar M. Hammad, Mohammed M. Al Gadban, Andrea J. Semler, Richard L. Klein

**Affiliations:** ^1^Department of Regenerative Medicine and Cell Biology, Medical University of South Carolina, Charleston, SC 29425, USA; ^2^Division of Endocrinology, Metabolism, and Medical Genetics, Department of Medicine, Medical University of South Carolina, Charleston, SC 29425, USA; ^3^Research Service, Ralph H. Johnson VA Medical Center, United States Department of Veterans Affairs, Charleston, SC 29401, USA

## Abstract

The physiological significance of sphingosine 1-phosphate (S1P) transport in blood has been debated. We have recently reported a comprehensive sphingolipid profile in human plasma and lipoprotein particles (VLDL, LDL, and HDL) using HPLC-MS/MS (Hammad et al., 2010). We now determined the relative concentrations of sphingolipids including S1P in the plasma subfraction containing lipoproteins compared to those in the remaining plasma proteins. Sphingomyelin and ceramide were predominantly recovered in the lipoprotein-containing fraction. Total plasma S1P concentration was positively correlated with S1P concentration in the protein-containing fraction, but not with S1P concentration in the lipoprotein-containing fraction. The percentage of S1P transported in plasma lipoproteins was positively correlated with HDL cholesterol (HDL-C) concentration; however, S1P transport in lipoproteins was not limited by the concentration of HDL-C in the individual subject. Thus, different plasma pools of S1P may have different contributions to S1P signaling in health and disease.

## 1. Introduction

Sphingolipids have been implicated in diseases such as cancer, obesity, and atherosclerosis; however, efforts addressing blood sphingolipids as biomarkers of disease or targets for therapeutics are still in their infancy. Sphingosine-1-phosphate (S1P) is a bioactive lipid that has been shown to play major roles in immunity, inflammation, and cardiovascular physiology [1–6]. S1P is found in plasma at relatively high concentrations (>200 nM) and is transported in blood bound both to lipoproteins (~65%) and albumin [7–9] with the bulk of the lipoprotein-associated S1P found in HDL (~54%), especially the smaller diameter HDL3 subfraction [10, 11]. Plasma HDL cholesterol levels correlate positively with those of plasma S1P [12], but HDLs do not appear to be merely inert carriers of S1P as the S1P constituent of HDL is biologically active and contributes to numerous metabolic effects of HDL [10, 11, 13–16]. 

We have recently reported a comprehensive sphingolipid profile in “normal” human plasma and lipoprotein particles (VLDL, LDL, and HDL) using HPLC-MS/MS [9]. As an extension of this study we now identify the nonlipoprotein (albumin)-associated versus the lipoprotein-associated sphingolipids. It is established that the concentration of S1P in plasma/serum is much higher than the half-maximal concentration of S1P needed to stimulate its receptors. Nevertheless, it was shown for instance that the inositol phosphate response mediated by S1P receptors was much smaller than the response expected from the total amount of S1P introduced to cells [8]. This response to exogenous S1P was markedly attenuated in the presence of lipoprotein-deficient serum, and was associated with the trapping of exogenous S1P [8]. Importantly, HDL and LDL showed a stronger activity for trapping S1P than lipoprotein-deficient serum [8]. As such, these findings suggest that the “tight” binding of S1P to lipoproteins may interfere with the S1P binding to its receptors and thereby attenuate the S1P-receptor-mediated cell responses.

The physiological significance of S1P transport in HDL versus bound to albumin has been debated and this has led to the suggestion that S1P may be atheroprotective when bound to HDL but proatherogenic when localized to the plasma protein fraction of blood bound to albumin [1, 7, 16–18]. Most importantly, the distribution of S1P between the plasma lipoprotein and protein fractions of blood is reportedly altered in patients with coronary artery disease [19]. We determined the concentrations of sphingolipids in total plasma, in the subfraction of plasma containing all the lipoprotein fractions, and in the plasma subfraction containing the remaining plasma proteins which were separated using ultracentrifugation of plasma. In addition, we investigated the distribution of S1P between the lipoprotein and plasma protein S1P pools and the influence of plasma lipoprotein and lipid concentrations on this distribution.

## 2. Materials and Methods

### 2.1. Study Subjects

Subjects aged 30–58 years were screened before participating in the study and those with known heart disease, kidney disease, diabetes, cancer, or serious current illness were excluded. Smokers, pregnant females, and subjects who take daily multivitamins or antioxidants were also excluded. A conventional lipid panel (total cholesterol (C), HDL-C, LDL-C, VLDL-C, and triglycerides) was determined in each subject (Cholestech LDX, Cholestech Corporation, Hayward, CA, USA). The plasma lipid and lipoprotein profiles for each subject are summarized in Table 1. Participating study subjects (*n* = 6) were asked to fast overnight for at least 10 h before collecting the blood sample for analysis. The study was approved by the Institutional Review Board (IRB) at the Medical University of South Carolina (MUSC) and proper consent was obtained from each subject.

### 2.2. Blood Sample Collection

Blood was collected in the presence of a lipoprotein preservative cocktail EDTA (0.1% w/v), chloramphenicol (20 *μ*g/ml), gentamycin sulfate (50 *μ*g/ml), epsilon aminocaproic acid (0.13% w/v), and dithiobisnitrobenzoic acid (0.04% w/v) to inhibit LCAT activity (final concentrations). Blood was then centrifuged (2,400 ×g, 20 min, 4°C) to obtain plasma. Plasma samples were stored at 4°C until analyzed further.

### 2.3. Conventional Plasma Lipoprotein Profiles

Plasma conventional lipid profiles were determined by Cholestech LDX (Cholestech Corporation, Hayward, CA, USA).

### 2.4. Separation of Lipoproteins from Plasma Proteins

The fraction of plasma containing all the lipoproteins (VLDL, LDL, and HDL) was separated from the plasma subfraction containing the nonlipoprotein, plasma proteins using isopycnic density ultracentrifugation. Plasma solvent density was adjusted to *d* = 1.225 g/ml with solid potassium bromide (KBr) and all the lipoproteins in plasma were isolated by preparative ultracentrifugation of the plasma samples (10 ml) using a type SW41Ti rotor (Beckman Coulter, Fullerton, CA, USA) spun at 41,000 rpm (288,000 ×g) for 40 h at 4°C in an Optima TM XL-100K ultracentrifuge. The floating lipoprotein subfraction in the ultracentrifugal supernatant was separated from the sedimented plasma lipoproteins in the subnatant after slicing each tube exactly 1.25 inch from the meniscus. The lipoprotein supernatant and plasma protein subnatant fractions were quantitatively harvested and the volume of each subfraction was adjusted to 10 ml using saline/EDTA (150 mM NaCl, 300 *μ*M EDTA, pH 7.4) and the fractions were stored at 4°C until used. Cholesterol levels in the total lipoprotein preparation in the ultracentrifugal supernatant and in the subnatant containing the nonlipoprotein plasma proteins were measured using gas chromatography as described previously [20]. Whole plasma and the ultracentrifugally prepared plasma subfractions were analyzed using electrophoresis in agarose gels to assess the purity of the subfractions (Beckman Paragon LIPOEPG, Beckman-Coulter, Fullerton, CA, USA).

### 2.5. Sphingolipid Extraction and Analysis

Analysis of endogenous sphingoid bases, sphingoid base 1-phosphates, ceramide, and sphingomyelin (SM) species were conducted in the Lipidomics Core Facility at MUSC as previously described [9]. Briefly, 100 *μ*l of each plasma sample, and the ultracentrifuge supernatant and subnatant subfractions of plasma were spiked with internal standards and the sphingolipid complement in each sample was quantitatively extracted as described previously [9]. Analyses of sphingolipids were performed by high-performance liquid chromatography-tandem mass spectrometry (LC-ESI-MS/MS) as described previously [9]. The equipment consisted of a Thermo Scientific Accela Autosampler and Quaternary Pump (Waltham, MA, USA). A Thermo Scientific Quantum Access triple quadrupole mass spectrometer equipped with an Electrospray Ion Source (ESI) operating in multiple reaction monitoring (MRM) positive ion mode was used. Chromatographic separations were obtained under a gradient elution of a Peeke Scientific (Redwood City, CA, USA), Spectra C8SR 150 × 3.0 mm, 3-*μ*m particle size column. Quantitative analyses were based on calibration curves generated by injecting known amounts of the target analytes and an equal amount of the internal standards. Final concentrations of analytes in samples were determined using the appropriate corrections for sample loss based on internal standard recovery calculations. Sphingolipids with no available standards were quantified using the calibration curve of its closest counterpart. The resulting data were then normalized to the volume of sample analyzed.

## 3. Statistics

Data were analyzed using a commercially available statistical analysis package (SigmaStat v3.0, SPSS, Chicago, IL, USA).

## 4. Results

We selectively recruited six subjects whose plasma lipid and lipoprotein levels (Table 1) were representative of normolipidemia (subjects 1–4), combined hyperlipoproteinemia (subject 5), and hypertriglyceridemia (subject 6). Most importantly, these subjects provided the potential to investigate the concentrations and distribution of sphingolipids over a broad range of plasma HDL-C (0.7–2.1 mM) concentrations.

**Figure 1:**
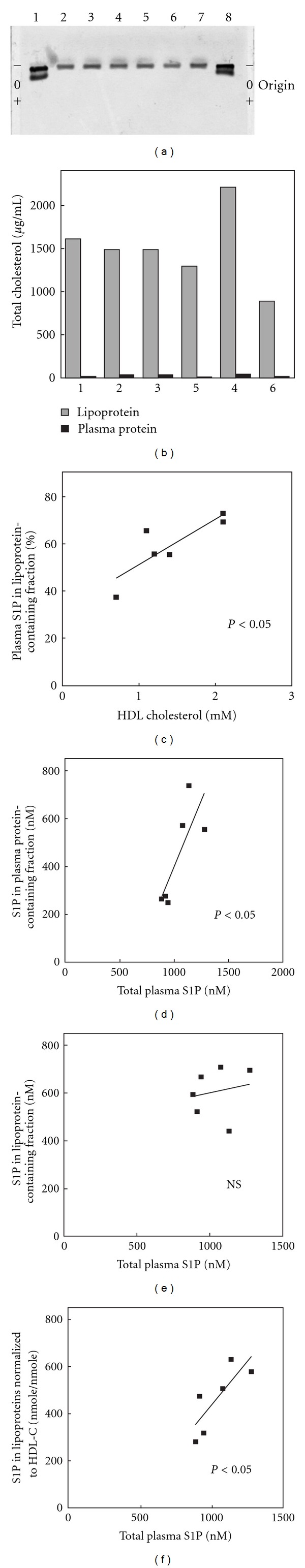
Analyses of human plasma sphingosine 1-phosphate (S1P) in lipoprotein- and protein-containing fractions. (a): agarose gel electrophoretogram of *d* > 1.21 g/ml plasma sub-fraction from each subject (lanes 2–7) with whole plasma (lane 1) and *d* < 1.21 g/ml plasma subfraction from a representative subject (lane 8) shown for reference. (b): total cholesterol concentration in the lipoprotein- and plasma protein-containing fractions from each subject determined by gas chromatography. (c): association between HDL cholesterol concentration and the percentage of S1P in plasma localized to the lipoprotein-containing fraction. (d): association between total plasma S1P concentration and the S1P concentration in the plasma protein-containing fraction. (e): association between total plasma S1P concentration and the S1P concentration in the ultracentrifugally isolated lipoprotein-containing fraction. (f): association between total plasma S1P concentration and the S1P concentration in the lipoprotein-containing fraction normalized by the HDL cholesterol concentration in the subject.

We determined the concentrations of sphingolipids in the fraction of plasma containing all the plasma lipoproteins distinct from plasma fraction containing the remaining plasma proteins. These two plasma fractions were prepared by ultracentrifugation of whole plasma to float all the lipoprotein classes to separate them from the remaining plasma proteins which sedimented to the tube bottom. There were no detectable lipid staining bands with mobility comparable to plasma lipoproteins in the ultracentrifuge subnatant fractions after electrophoresis of the ultracentrifuge super- and subnatants in agarose gels (Figure 1(a)) confirming that plasma lipoproteins had been quantitatively removed from the fraction containing the plasma proteins. We also determined the concentrations of total cholesterol in each plasma fraction using gas chromatography (Figure 1(b)). Cholesterol levels in the plasma protein-containing fraction averaged only 1.8 ± 0.6 percent of the total cholesterol in plasma. Collectively, these data suggest that using the methods described, lipoproteins (lipoprotein cholesterol) were quantitatively removed from the plasma fraction containing the remaining plasma proteins.

The concentrations of the molecular species of ceramides measured in whole plasma, in the fraction of plasma containing all the plasma lipoproteins, and the plasma fraction containing the plasma proteins are shown in Table 2. Ceramide concentrations in the lipoprotein-containing fraction averaged 96 ± 3% of total plasma ceramide content in agreement with their role as an integral lipoprotein structural constituent and further support our conclusion that we quantitatively separated lipoproteins from the remaining plasma proteins. The concentrations of the ceramide species in the lipoprotein- and plasma protein-containing fractions averaged 97 ± 5% of the total ceramide concentration in plasma in support of our conclusion that recovery of sphingolipid in the plasma fractions after ultracentrifugation was quantitative. The ceramides present in highest concentration were the C_24_, C_24:1_, C_20_, and C_22_ species in agreement with our previous reports [9, 11]. We also analyzed the plasma concentrations of the SM species which averaged (mean ± SD, *μ*M) 10.7 ± 5.4 (C_14_-SM), 89.7 ± 26.9 (C_16_-SM), 5.8 ± 1.2 (C_18_-SM), 2.3 ± 0.9 (C_18:1_-SM), 6.1 ± 1.6 (C_20_-SM), 1.5 ± 0.6 (C_20:1_-SM), 10.8 ± 2.0 (C_22_-SM), 7.9 ± 2.6 (C_22:1_-SM), 8.4 ± 2.3 (C_24_-SM), and 17.1 ± 4.2 (C_24:1_-SM). SM species distribution in the lipoprotein-containing fraction averaged 96.4 ± 1.5% of total plasma SM in agreement with our findings for ceramide species distribution. This data provides additional support of our conclusion that the separation of lipoproteins from the plasma protein fraction after ultracentrifugation was quantitative.

The concentrations of sphingoid bases, sphingosine and dihydrosphingosine, and their 1-phosphates (S1P and dhS1P) measured in whole plasma, in the fraction of plasma containing all the plasma lipoproteins, and in the plasma fraction containing the plasma proteins are summarized in Table 3. The percentage of S1P concentration in plasma localized to the lipoprotein-containing fraction was significantly, positively correlated (*R* ^2^ = 0.7117; *P* < 0.05) with HDL cholesterol concentration in the six subjects (Figure 1(c)). The total concentration of S1P in plasma varied significantly among the six subjects studied (Table 3). We wished to determine if increases in S1P mass in plasma were transported primarily in lipoproteins or were localized to the plasma protein-containing fraction. Increasing concentrations of S1P in plasma were significantly and positively correlated with the concentrations of S1P in the plasma protein-containing fraction (Figure 1(d)) (*R* ^2^ = 0.65; *P* < 0.05), but not with the S1P concentrations in the lipoprotein-containing fraction (Figure 1(e)). Because the bulk of the lipoprotein-associated S1P is found in HDL and HDL concentration in the six subjects investigated varied by almost 2.9-fold (Table 1), we investigated if the S1P concentration in the lipoprotein-containing fraction in each subject was limited by the HDL concentration in the subject. Therefore, we normalized the S1P concentration in the lipoprotein-containing fraction by the HDL cholesterol concentration in the subject (Table 3). There was a significant, positive association (*R* ^2^ = 0.6551; *P* < 0.05) between total S1P concentration in plasma with the “normalized” amount of S1P in the lipoprotein-containing fraction when the concentration of S1P was normalized by the HDL cholesterol concentration in the subject (Figure 1(f)).

## 5. Discussion

We investigated the associations of plasma lipid and lipoprotein concentrations with the total plasma concentrations of ceramides and sphingoid bases, sphingosine, and dihydrosphingosine, and their 1-phosphates. We also determined the distribution of these sphingolipids between the pool localized to plasma lipoproteins compared to that found in the remaining plasma proteins mainly bound to albumin. We determined that S1P concentration in plasma is highly variable and determined further that S1P in plasma was nonuniformly distributed between the plasma lipoprotein and plasma protein pools of S1P in the six subjects investigated. We determined further that increasing concentrations of S1P in plasma were localized primarily to the plasma protein-containing fraction (Figures 1(d) versus 1(e)) in subjects exhibiting a broad range of plasma lipid concentrations (Table 1).

The results of our studies in these select subjects confirmed and extended those reporting previously [12] that S1P concentrations in the lipoprotein-containing fraction of plasma were positively correlated with HDL cholesterol concentrations (Figure 1(c)). Because HDL is the primary lipoprotein which transports S1P, and because HDL-C concentration varied widely in the six subjects (Table 1), we also normalized the levels of S1P in the lipoprotein-containing fraction to the HDL-C concentration in each subject (Figure 1(f)). We determined that this “normalized” concentration of S1P transported in the lipoprotein-containing fraction was positively correlated to the total S1P concentration in plasma, which suggests that the S1P content of HDL is variable and that S1P transport in lipoproteins was not limited by the concentration of HDL-C in the individual subject. In fact, we have demonstrated previously that per particle, the larger VLDL particle contains the highest content of S1P, and the smallest lipoprotein particle, HDL3, contains higher S1P levels than LDL particles [9]. Our current data may allow subsequent research on mechanisms that mediate binding of the different S1P carriers to S1P receptors, knowing that only 5% of HDL particles carry S1P molecules [21].

Recent studies have reported that HDL-associated S1P is bound specifically to apolipoprotein M (apoM) and, furthermore, is transported selectively in apoM-containing HDL particles [22]. We did not measure apoM concentration or distribution in this select subject set and thus cannot infer if differences in apoM metabolism between the subjects were associated with the observed differences in S1P metabolism. Alternatively, we [9, 11] and others [8, 10] have determined that S1P levels in HDL are significantly higher in the smaller sized HDL3 subfraction compared to S1P levels in HDL2. The smaller HDL3 particles were shown also to be enriched in apoM [23]. Despite the very strong evidence from apoM knockout and apoM-transgenic mice that apoM determines S1P concentrations in plasma and HDL [22, 23], there was no statistically significant correlation between S1P and apoM concentrations in human patients with different monogenic disorders of HDL metabolism [23]. Thus, the relative concentrations of HDL subfractions may have influenced the distribution of S1P in the individual subjects. 

More recently, the crystal structure of a main S1P G protein-coupled receptor, S1P1, was revealed, and it was found that extracellular access to the binding pocket of this receptor is occluded by the aminoterminus and extracellular loops of the receptor [24]. Interestingly, access is gained by ligands entering laterally between helices I and VII within the transmembrane region of the receptor [24]. In the context of previous findings, we postulate that effects of HDL-bound S1P could be related to (1) HDL particle size and the ability to modulate cell lipid rafts and comprised receptors, (2) S1P content of HDL particles being higher in the smaller HDL particles, and (3) HDL role as scavenger/reservoir for biologically active lipids including S1P. 

A confounding factor in recent studies addressing the cardioprotective role of S1P levels in HDL is the assumption that 30% of S1P is recovered in the lipoprotein-deficient serum [25]. As shown in Table 3, S1P distribution in the plasma protein-containing fraction approximated 30% in only three of the six subjects studied. Thus, even in this limited number of subjects, S1P distribution in 50% of the subjects does not support this often cited assumption. Figure 1(e) clearly shows that there is no significant association between total plasma S1P and S1P concentration in plasma lipoprotein-containing fraction, which suggests a crucial pathophysiological role of the S1P bound to plasma proteins. Furthermore, the procedure in which apolipoprotein B-depleted plasma is prepared and S1P concentration is subsequently determined in the “HDL-containing” fraction as performed in two recent studies [21, 26] also does not discriminate between HDL- and albumin-associated S1P. Thus, when this methodology is employed, assumptions regarding S1P distribution in plasma may result in erroneous estimates.

We have shown previously that the secretion of plasminogen activator inhibitor-1 (PAI-1) from cultured adipocytes is significantly increased when the cells were incubated with increasing S1P concentration in the media regardless of whether the S1P is bound to HDL or transported by albumin [11]. Previous studies have determined that triglyceride level was independently related to plasma PAI-1 activity level in both subjects with hypertriglyceridemia and in age-matched normotriglyceridemic subjects [27]. The factor(s) contributing to this phenomenon are still unclear. In this limited study, plasma S1P concentrations were increased in the two subjects exhibiting elevated plasma triglyceride levels. Clearly, additional studies are warranted in this area.

In summary, we determined that S1P concentration in plasma is highly variable, S1P in plasma is nonuniformly distributed between the plasma lipoprotein and plasma protein pools of S1P and increasing concentrations of S1P in plasma are localized primarily to the plasma protein-containing fraction. We also determined that the S1P content of HDL, the major lipoprotein carrier of S1P, is variable and that S1P transport in lipoproteins is not limited by the concentration of HDL-C. The data further show that there is no significant association between total plasma S1P and S1P concentration in plasma lipoprotein-containing fraction, which suggests a crucial pathophysiological role of the S1P bound to plasma proteins.

## Figures and Tables

**Table 1:** Plasma lipid and lipoprotein profiles in study subjects.

Subject	Gender	Age^a^	Total cholesterol (TC)	Total triglycerides	LDL-C	HDL-C	VLDL-C	TC/HDL-C
1	Female	56	4.1 (156)^b^	0.6 (50)	1.7 (66)	2.1 (80)	0.3 (10)	2.0
2	Male	30	4.2 (160)	1.4 (129)	2.1 (80)	1.4 (54)	0.7 (26)	3.0
3	Female	42	4.3 (165)	1.3 (117)	1.6 (60)	2.1 (81)	0.6 (23)	2.0
4	Female	48	4.5 (174)	1.8 (165)	2.6 (99)	1.1 (42)	0.9 (33)	4.1
5	Female	54	6.0 (231)	2.4 (220)	3.6 (138)	1.2 (48)	1.1 (44)	4.8
6	Male	58	2.5 (97)	4.5 (408)	0.5 (19)^c^	0.7 (28)	1.3 (50)	3.5

^a^Years.

^b^mM (mg/dL).

^c^Lipoprotein cholesterol determined directly.

**Table:** (a)

Subject	Concentration (nM)												
	C14-Cer	C16-Cer	C18-Cer	C18:1-Cer	C20-Cer	C20:1-Cer	C22-Cer	C22:1-Cer	C24-Cer	C24:1-Cer	C26-Cer	C26:1-Cer	dhC16-Cer
1	118	445	87	45	282	14	340	83	2498	1090	112	46	55
2	48	158	80	26	279	13	377	85	2773	1035	117	45	53
3	77	146	68	35	297	8	322	62	2548	728	70	21	53
4	50	250	84	33	156	10	383	78	2994	1325	91	39	62
5	170	548	138	80	420	20	486	111	3975	1476	181	66	103
6	77	182	114	22	493	11	465	83	3001	1084	88	46	36

**Table:** (b)

Subject	Concentration (nM)												
	C14-Cer	C16-Cer	C18-Cer	C18:1-Cer	C20-Cer	C20:1-Cer	C22-Cer	C22:1-Cer	C24-Cer	C24:1-Cer	C26-Cer	C26:1-Cer	dhC16-Cer
1	121	459	89	66	455	20	405	99	3388	1354	147	57	42
2	57	135	76	24	274	15	394	85	2347	1046	86	41	49
3	71	161	99	38	352	13	311	63	2351	687	61	22	52
4	62	332	56	31	214	10	383	80	2570	1193	88	33	64
5	183	774	116	66	537	27	598	129	4819	1786	201	71	113
6	88	178	121	24	540	13	477	84	3130	1106	94	50	48

**Table:** (c)

Subject	Concentration (nM)												
	C14-Cer	C16-Cer	C18-Cer	C18:1-Cer	C20-Cer	C20:1-Cer	C22-Cer	C22:1-Cer	C24-Cer	C24:1-Cer	C26-Cer	C26:1-Cer	dhC16-Cer
1	ND	5	8	3	6	ND	27	2	28	16	1.5	0.3	1.3
2	ND	8	9	3	10	0.6	28	6	39	32	2.0	0.8	1.3
3	1.6	11	12	1	4	0.1	24	2	19	13	1.4	ND	1.7
4	ND	2	4	1	3	0.4	25	2	17	22	1.2	ND	0.5
5	2.7	10	17	1	13	0.9	38	7	75	36	1.5	0.6	0.9
6	7.5	15	15	4	14	0.8	27	5	31	28	2.1	0.4	3.5

ND: not detected, Cer: ceramide.

**Table:** (a)

Subject	Concentration (nM)			
	dhSph	dhS1P	Sph	S1P
1	13	157	8	939
2	19	114	19	1072
3	10	132	18	884
4	14	138	20	913
5	7	171	23	1272
6	19	147	24	1132

**Table:** (b)

Subject	Concentration (nM)				S1P normalized to HDL-C
	dhSph	dhS1P	Sph	S1P	(nmole/mmole)
1	6	33	11	669	32
2	19	21	15	710	51
3	4	31	9	595	28
4	3	34	11	523	48
5	17	26	12	696	58
6	19	12	18	442	63

**Table:** (c)

Subject	Concentration (nM)				
	dhSph	dhS1P	Sph	S1P	S1P in plasma protein fraction (%)
1	7	105	7	248	27
2	8	133	8	571	45
3	8	111	10	263	31
4	7	79	10	215	34
5	11	150	7	555	44
6	8	117	19	919	63

Sph: sphingosine, dhSph: dihydrosphingosine, their 1-phosphates S1P and dhS1P.

## Abbreviations

S1P: sphingosine 1-phosphate
VLDL: very low-density lipoproteins
LDL: low-density lipoproteins
HDL: high-density lipoproteins
SM: sphingomyelin
C: total cholestero
dhS1P: dihydrosphingosine 1-phosphate
PAI-1: plasminogen activator inhibitor-1.
